# Study on the Discrimination between Citri Reticulatae Pericarpium Varieties Based on HS-SPME-GC-MS Combined with Multivariate Statistical Analyses

**DOI:** 10.3390/molecules23051235

**Published:** 2018-05-22

**Authors:** Yuying Zheng, Xuan Zeng, Wei Peng, Zhong Wu, Weiwei Su

**Affiliations:** Guangdong Engineering & Technology Research Center for Quality and Efficacy Reevaluation of Post-Market Traditional Chinese Medicine, Guangdong Key Laboratory of Plant Resources, School of Life Sciences, Sun Yat-sen University, Guangzhou 510275, China; vicky_0224@126.com (Y.Z.); zengx6@mail2.sysu.edu.cn (X.Z.); pweiyu929@126.com (W.P.); wuzhong1962@126.com (Z.W.)

**Keywords:** Citri reticulatae pericarpium, volatile compounds, HS-SPME-GC-MS, multivariate statistical analyses

## Abstract

Citri reticulatae pericarpium (CRP), the dried pericarps of *Citrus reticulata* Blanco and its cultivars, has been widely used in drugs and foods in China for centuries. In this study, an accurate and feasible analytical method based on HS-SPME-GC-MS coupled with multivariate statistical analyses was developed to comprehensively compare volatile compounds of pericarps derived from *Citrus reticulata* “Chachi” (“Guangchenpi” in Chinese, GCP) and other cultivars of *Citrus reticulata* Blanco (“Chenpi” in Chinese, CP). Principal component analysis, hierarchical cluster analysis, and orthogonal partial least-squares-discrimination analysis were performed to extract meaningful attributes from volatile profiles based on GC-MS data. Results indicated that samples from GCP and CP could easily be differentiated, and seven potential chemical markers were screened for the quality control of CRP. This study illuminated the volatile profile in CRP, and provides a practical method for the authentication of CRP varieties.

## 1. Introduction

Citri reticulatae pericarpium (CRP) is the sun-dried pericarp of mature fruits of *Citrus reticulata* Blanco (commonly known as tangerine) and its cultivars, and is widely used in drugs and foods in China, involved in more than 10% of all formulae in the Chinese Pharmacopoeia (2015 edition) [1]. *Citrus reticulata* Blanco grows throughought southern China, and includes various cultivars, such as ”Chachi”, ”Unshiu”, ”Zhuhong”, ”Ponkan”, ”Suavissima”, etc. Generally, the pericarp of Citrus reticulata”Chachi” from Xinhui County (Guangdong province, China), called “Guangchenpi” (GCP) in Chinese, has always been well-regarded as the best national product with respect to geo-herbalism [2]. Pericarps derived from other cultivars are collectively called “Chenpi” (CP) in Chinese, and their origins are diverse in the market [3]. As GCP and CP have quite similar appearances and flavor characteristics, it is difficult to discriminate between them and this could easily lead to economic adulteration. Actually, due to its premium price, GCP is prone to being superseded by cheaper or inferior variants so as to get a higher profit margin in the market. Therefore, there exists significant interest in the development of accurate methods differentiating GCP and CP.

Chemical profiling has been increasingly applied in the authentication of traditional Chinese medicines (TCMs) [4,5,6]. Until now, flavonoids contained in CRP have been analyzed and reported in many studies [7,8,9], while volatile constituents have been rarely focused on. Given that volatile bioactive compounds possess potent efficacy, it is meaningful to compare them in GCP and CP. Extraction is a crucial procedure in the analysis of volatile compounds in CRP, and several methods of doing so (hydro-distillation, carbon dioxide supercritical fluid extraction) have been reported [10,11,12]. However, these methods are time- and material- consuming. In recent years, headspace solid-phase microextraction (HS-SPME), which has been proven to be a rapid, simple, and convenient sampling method, has increasingly been used in the analysis of volatile compounds [13,14].

In this study, an accurate and robust method based on HS-SPME-GC-MS and multivariate statistical analyses was developed to discriminate between GCP and CP on the basis of potential volatile compounds. Multivariate statistical methods, including principal component analysis (PCA), hierarchical cluster analysis (HCA), and orthogonal partial least-squares-discrimination analysis (OPLS-DA), were employed to screen potential chemical markers for differentiating GCP and CP. This study facilitated the profiling of volatile constituents of CRP, and provides a practical method for the authentication of CRP varieties.

## 2. Results and Discussion

### 2.1. Optimization of SPME Conditions

**Figure 1:**
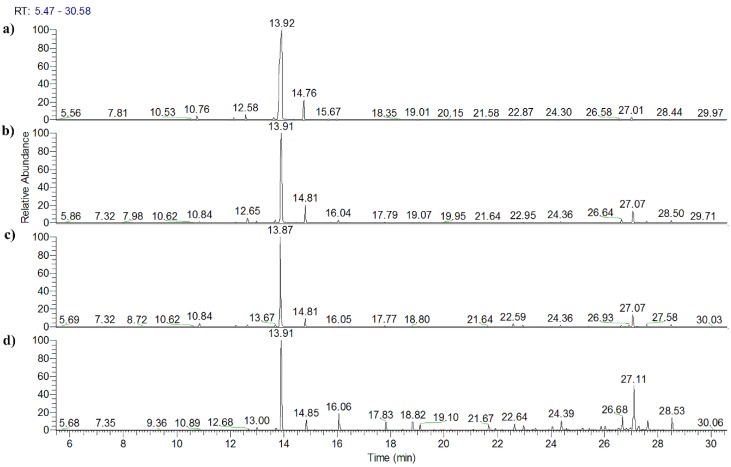
GC-MS typical total ion chromatograms (TICs) of volatile compounds in the “Chenpi” (CP) sample (S5). (**a**) Extracted through hydro-distillation. (**b**) Extracted through headspace solid-phase microextraction (HS-SPME) at 30 °C for 10 min. (**c**) Extracted through HS-SPME at 50 °C for 10 min. (**d**) Extracted through HS-SPME at 50 °C for 30 min.

Unlike exhaustive extraction through hydro-distillation, the volatile profiles of extracts obtained using SPME deeply depend on fiber type, sampling temperature, and sampling time [10]. In order to evaluate the feasibility of SPME conditions, volatile profiles of a CP sample (S5) were obtained through SPME, and compared with those that flowed from hydro-distillation. The hydro-distillation process was conducted in accordance with the procedure described in the Chinese Pharmacopoeia [15]. The volatile profile of S5 extracted through hydro-distillation is shown in Figure 1a. Monoterpenes were found to be the predominant compounds, including d-limonene, γ-terpinene, α-pinene, β-pinene, etc. The volatile profiles extracted through HS-SPME were basically consistent with that of the hydro-distillation method. Furthermore, when the sampling temperature was 50 °C, the components with higher boiling points received a relatively high response (shown in Figure 1b,c). As extraction time increased, the percentage of monoterpenes (components with lower boiling points) decreased, and the proportion of components with higher boiling points increased (shown in Figure 1d). The percentage peak areas of the two main monoterpenes (d-limonene and γ-terpinene) and the two sesquiterpenes (d-germacrene and α-farnesene), obtained using various extraction methods and parameters, are presented in Table 1. As shown in previous studies [16,17], SPME can be used for quantitation in a non-equilibrium state. In order to obtain more comprehensive information about the volatile compounds of CRP, SPME conditions were selected so as to obtain a relatively high response for components with higher boiling points, which were proportionally low in CRP. The reproducibility of the method was evaluated through the determination of five replicates of a specified sample. The relative standard deviation (RSD) was found to oscillate between 6% and 14% (generally close to or lower than 10%). Acquired results showed that the conditions for extraction and GC-MS analysis were robust and reliable.

### 2.2. Volatile Compounds

A total of 51 volatile compounds, mainly monoterpenes and sesquiterpenes, were identified in the CRP extract processed by SPME. The detailed information is shown in Table S1. Results were consistent with reported data obtained through hydro-distillation and other methods [11]. Meanwhile, HS-SPME provided an easier sampling procedure in a shorter time (30 min). As shown in Table 2, 21 peaks, which were simultaneously detected in all 54 samples, were finally selected, and the peak-area ratios relative to that of n-tridecane were imported to form a dataset. Collectively, they made up an average of 85.2% of the total peak area, thus covering the majority of the information contained in the volatile profiles. Based on a previous report [18], volatile components which appeared in minute quantities were excluded for better repeatability of quantification.

### 2.3. Discrimination Between CP and GCP Based on Multivariate Analysis

**Figure 2:**
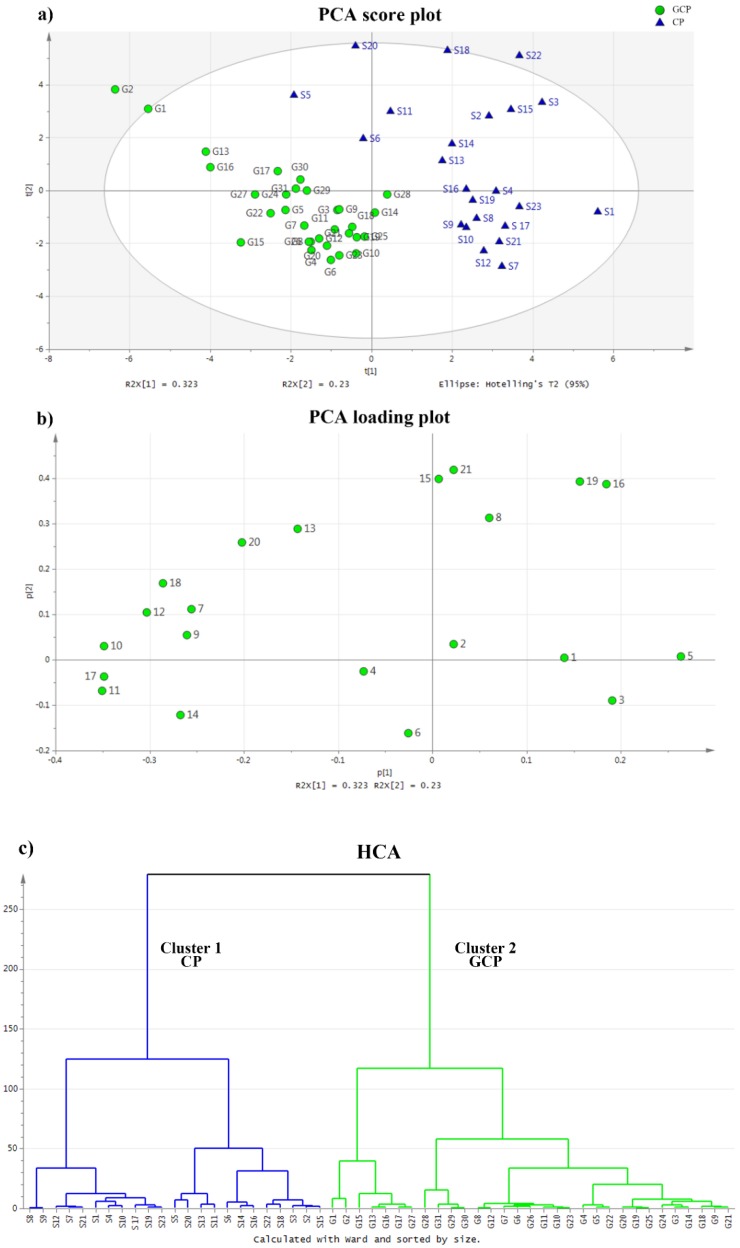
Multivariate data analysis of Citri reticulatae pericarpium (CRP). (**a**) Score plot of the principal-component-analysis (PCA) result. The variances accounted by the first principal component (PC1) and the second principal component (PC2) were 32.3% and 23.0%, respectively. Circles represent “Guangchenpi” (GCP) samples, and triangles represent CP samples. (**b**) Loading plot of the PCA result (PC1 vs. PC2). (**c**) Dendrograms of the hierarchical-cluster-analysis (HCA) result.

To investigate the chemical variation in volatile compounds based on GC-MS data, both unsupervised and supervised classification methods were employed. Unsupervised PCA analysis converted the original variables into new irrelevant variables (principal components, PCs). In this way, reduced dimensionality of the data was obtained while still preserving information from the original dataset. Thus, PCA revealed trends in the dataset, such as groupings and clusters based on chemical similarities or differences. In the presented study, four principal components accounting for 83.8% (R^2^X) of the total variance were considered significant (PC1 described 32.3% of the sample variability, and PC2 described 23%). The predictive ability of the model (Q^2^) was 68.8%, which indicated that it was a good model (Q^2^ ≥ 0.50). The PCA score plot and loading plot are shown separately in Figure 2a,b, respectively. The score plot (PC1/PC2) displayed the relationship between CP and GCP, and the loading plot showed variables that contributed to the score. Both domains (CP and GCP) were clearly separated from each other. The variables which possessed the greatest influence on classification were located at the furthermost position from the main cluster of variables [33]. HCA was performed based on four PCs from the above PCA model, and displayed relationships between various CRP samples in the form of a dendrogram, calculated using Ward’s minimum-variance method (shown in Figure 2c). All samples could be clearly divided into two clusters; all CP samples were classified into Cluster I (left), and all GCP samples were classified into Cluster II (right).

**Figure 3:**
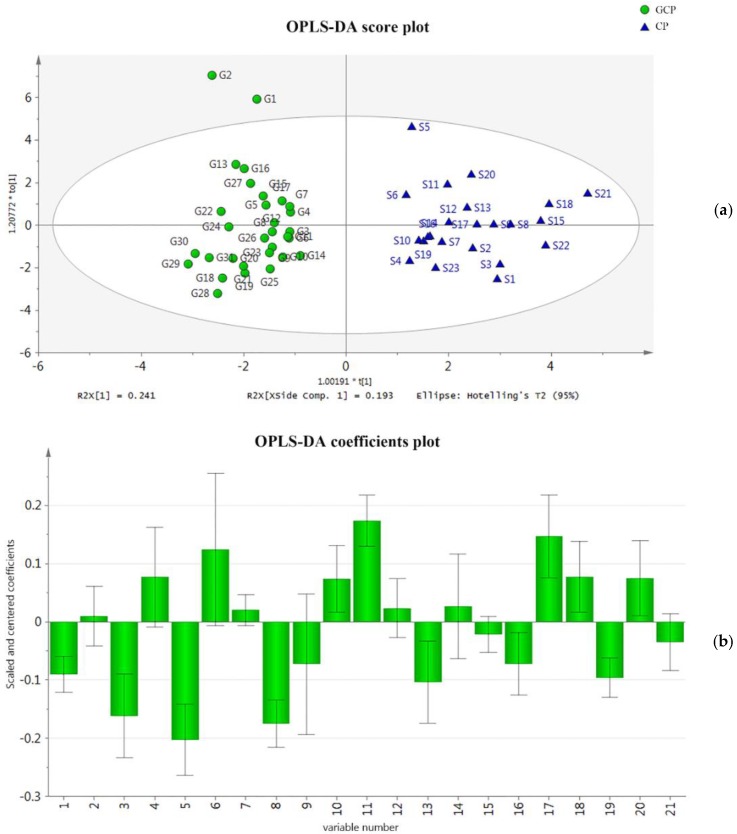
Orthogonal partial least-squares-discrimination analysis (OPLS-DA) models for the classification of CRP. (**a**) Score plot of the OPLS-DA results. Circles represent GCP samples, and triangles represent CP samples. (**b**) Plot of the coefficients for the OPLS-DA. The constant not displayed in the plot was 1.150.

Supervised OPLS-DA was applied in the discrimination, and also to define the most significant contributors toward discrimination. OPLS-DA extended a regression of the PCA, and used the class membership (as Y variables) to maximize variation, introducing an orthogonal signal correction filter to separately handle systematic variation correlated with or uncorrelated with the Y variable. Therefore, OPLS-DA had better discriminant ability than PCA for samples with larger intra-class divergence [34]. In the presented study, CP and GCP samples were clearly separated in the OPLS-DA score plot (shown in Figure 3a). The R^2^X of the OPLS-DA model was 0.633, indicating that 63.3% of the variation in the dataset could be modeled by the selected components. The R^2^Y was 0.878, indicating that the model was well fitted. The Q^2^ was 0.826, indicating a good predictive ability. The root-mean-square error of cross-validation (RMSECV) was used to evaluate the deviation between predicted and observed Y values, by summarizing the cross-validation residuals of observations in the dataset, and had a measurement unit of 0.164. A plot of the coefficients (shown in Figure 3b) displayed regression coefficients related to scaled and centered X-variables, which enabled the OPLS model to be rewritten as a regression model. This scaling of the data made the coefficients comparable, expressing how strongly the Y-variable was correlated with the systematic part of each X-variable. With the established OPLS-DA model, more sample data could be imported for discrimination.

To screen potential chemical markers that contributed to the differences between CP and GCP samples, seven compounds with variable importance in projection (VIP) scores greater than 1.2 were listed (shown in Table 3), demonstrating that they possessed the most influence on the discrimination between both types of CRP.

## 3. Materials and Methods

### 3.1. Materials

A total of 54 batches (23 batches of CP, and 31 batches of GCP) of mature tangerine fruits of various cultivars were collected from the main producing districts throughout China, between October 2016 and December 2016 (Table S2). They were all authenticated by Professor Wen-bo Liao (Sun Yat-sen University, China) according to their morphological characteristics. The samples were stored at the School of Life Sciences, Sun Yat-sen University, China.

After the fruits were washed, their flesh was manually removed. Then, their pericarps were removed according to criteria in the Chinese Pharmacopoeia. Finally, they were dried in sunless condition once impurities were discarded and stored under dry conditions.

### 3.2. Hydro-distillation Extraction

According to the Chinese Pharmacopoeia [15], 50 g of sample powder (S5) was dissolved in 500 mL of distilled water, and then subjected to hydro-distillation for 5 h in a standard extractor. Neat essential oils were collected and used for subsequent GC–MS analysis.

### 3.3. HS-SPME Extraction

Before extraction, the pericarps were powdered using a mill, and passed through a 60-mesh sieve (aperture size of 0.25 mm). Individual samples (20 mg) were accurately weighed, and placed in a 10-mL crimp-top headspace vial for SPME. After adding 5 μL of n-tridecane (0.1646 mg/mL dissolved in methanol) as an internal standard, the above-mentioned vials were sealed with magnetic caps and polytetrafluoro-ethylene (PTFE)-coatedsilicone septa.

The volatile compounds were sampled by HS-SPME with 50/30 μm divinylbenzene/carboxen fibre/polydimethylsiloxane (DVB/CAR/PDMS), supplied by Anpel Laboratory Technologies (Shanghai, China). Incubation and extraction were performed at 50 °C for 5 min and 30 min, respectively, under continuous agitation. Desorption was carried out in the injector at 220 °C for 10 min. Incubation, extraction, and desorption of volatile compounds was automatically performed by a TriPlus™ RSH autosampler (Thermo Fisher Scientific, Milan, Italy).

### 3.4. Analysis of Volatile Compounds through GC-MS

Essential oils extracted after hydro-distillation, as well as all SPME samples, were run on a GC-MS system. GC-MS analysis was carried out using a Trace GC Ultra gas chromatograph coupled with a triple quadrupole mass spectrometer (TSQ Quantum XLS, Thermo Fisher Scientific, Milan, Italy). Injections were performed in split mode (1:50), and volatile compounds were chromatographed on a TG-5SILMS column (30 m × 0. 25 mm × 0. 25 μm) provided by Thermo Fisher Scientific (Milan, Italy), with helium as the carrier gas at a constant flow of 1.0 mL/min. The oven temperature was maintained at 40 °C for 3 min, and then increased to 200 °C at a rate of 5 °C/min, before being subsequently increased to 250 °C at a rate of 10 °C/min, and then finally held at 250 °C for 3 min. The inlet and ion source temperatures were both set at 230 °C, and MS was scanned at 70 eV in the electronic ionization mode. The mass spectra were acquired using the full-scan-monitoring mode, with a mass-scan range of *m*/*z* 29–448, equipped using the Xcalibur software (v. 2.2, Thermo Fisher Scientific).

### 3.5. Data Analysis and Statistics

Compounds were tentatively identified by comparing the mass spectra and linear retention indices (LRI) with the NIST (version 11) mass spectral database (National Institute of Standards and Technology, Washington, DC, USA). The mixture of a homologous series of n-alkane standards (C_7_–C_30_) dissolved in n-hexane, employed for LRIs, was supplied by Supelco (Bellefonte, PA, USA), and analyzed under the same conditions as the samples. Quantitative data of the identified compounds, which were used for statistical analysis, were based on the peak-area ratios relative to that of n-tridecane (internal standard).

Multivariate analyses (PCA, HCA, and OPLS-DA) were performed using the SIMCA-P version 13.0 software (Umetrics, Malmö, Sweden) to evaluate relationships on the basis of similarities or differences between groups of multivariate data. Unit-variance scaling was applied in data preprocessing for both PCA and OPLS-DA. PCA results were displayed in the form of score plots and loading plots. HCA was performed based on principal components from the PCA model, and distances between 54 samples were calculated using Ward’s minimum-variance method, with results presented as a dendrogram. OPLS-DA was conducted using class information as the Y-variable, and results were shown in the form of score plots and coefficient-coded loading plots [34]. The quality of the model was estimated by parameters from a seven-round internal cross validation: R^2^X showed how much of the variation in the X variable could be explained by the selected components, R^2^ Y described how well the model was fitted, and Q^2^ showed the predictability of the model. R^2^ and Q^2^ values ranged from 0 to 1, where 1 indicated perfect fitness. In the OPLS-DA model, the root-mean-square error of cross-validation (RMSECV) also described the predictive power of the model, but did so by comparing the originally observed Y-variable and the predicted Y-variable using the measurement unit [35]. The contribution of variables to the analysis was explained using variable importance in projection (VIP) scores. VIP scores were a weighted sum of squares of PLS weights, with scores larger than 1 indicating variables were important to the model.

## 4. Conclusions

In the presented study, an accurate and feasible analytical method based on HS-SPME-GC-MS coupled with multivariate statistical analyses was developed for objective discrimination between CRP varieties. Informative chemical profiles of 54 batches of CRP with 11 origins were obtained and employed in multivariate statistical analyses, including PCA, HCA, and OPLS-DA. Samples from GCP and CP were found to be easily differentiated, and seven potential chemical markers were screened. This study provides a practical method for the authentication of CRP varieties, and results from the comparisons of volatile chemical profiles will facilitate further understanding of their differences with respect to pharmacology and pharmacokinetics.

## Figures and Tables

**Table 1:** Main terpenes identified in the analyzed “Chenpi” (CP) sample (S5) using various extraction methods and parameters.

Retention Time (RT) (min)	Compound	Peak Area (%)			
		Hydro-Distillation	Solid-Phase Microextraction (SPME) at 30 °C for 10 min	SPME at 50 °C for 10 min	SPME at 50 °C for 30 min
13.84	d-Limonene	81.52	61.46	52.35	28.09
14.77	γ-Terpinene	7.44	8.37	4.51	3.05
26.60	d-Germacrene	0.33	1.94	1.18	4.22
27.03	α-Farnesene	1.11	7.18	8.44	14.26

**Table 2:** Identification of volatile compounds in Citri reticulatae pericarpium (CRP).

No. ^a^	Compound	RT ^b^ (min)	LRI ^c^	Reference	Relative Area ^d^	
					“Guangchenpi” (GCP) (n = 31)	CP (n = 23)
1	α-Pinene	10.83	936	[19]	0.26 ± 0.14	0.41 ± 0.24
2	β-Pinene	12.19	977	[20]	0.25 ± 0.12	0.27 ± 0.15
3	β-Myrcene	12.62	990	[21,22]	0.35 ± 0.16	1.06 ± 0.88
4	*o*-Cymene	13.65	1022	[23]	1.33 ± 0.66	1.02 ± 0.53
5	d-Limonene	13.84	1028	[21,22,24]	24.29 ± 7.45	55.61 ± 26.24
6	γ-Terpinene	14.77	1058	[25]	8.55 ± 3.43	6.77 ± 3.95
7	trans-4-Thujanol	15.04	1066	[21]	0.97 ± 1.24	0.46 ± 0.29
8	2-Cyclohexen-1-ol, 1-methyl-4-(1-methylethyl)-, cis-	15.98	1096	[26]	5.51 ± 2.83	11.92 ± 6.29
9	trans-p-Mentha-2,8-dienol	16.64	1119	[22]	0.90 ± 0.45	0.67 ± 0.27
10	4-Terpineol	18.35	1177	[27]	2.69 ± 0.90	1.37 ± 0.87
11	p-Cymen-8-ol	18.49	1181	[21]	0.89 ± 0.28	0.26 ± 0.14
12	α-Terpineol	18.72	1189	[21]	10.22 ± 4.74	6.83 ± 2.50
13	Perilla aldehyde	21.00	1271	[28]	1.34 ± 0.56	1.50 ± 0.68
14	Carvacrol	21.57	1291	[29]	3.35 ± 1.23	2.06 ± 2.81
15	Copaene	23.97	1383	[30]	1.02 ± 0.54	1.57 ± 1.20
16	β-Cubebene	24.31	1396	[30]	0.70 ± 0.35	2.76 ± 2.26
17	Benzoic acid, 2-(methylamino)-, methyl ester	24.55	1406	[31]	40.07 ± 10.44	15.60 ± 10.47
18	Caryophyllene	25.11	1428	[30]	3.41 ± 1.96	1.61 ± 1.37
19	d-Germacrene	26.60	1489	[25]	0.51 ± 0.35	2.72 ± 2.42
20	α-Farnesene	27.03	1507	[32]	11.41 ± 5.14	8.72 ± 8.31
21	Cadinene	27.55	1529	[30]	1.71 ± 0.99	2.85 ± 2.24

^a^ Numbers represent the compound index for the principal-component-analysis (PCA) loading plot shown in Figure 2b. ^b^ Retention time (min). ^c^ Linear retention indices. ^d^ Peak-area ratios relative to n-tridecane (internal standard).

**Table 3:** List of potential chemical markers.

No.	RT (min)	Compounds	Variable Importance in Projection (VIP) Scores
1	18.49	p-Cymen-8-ol	1.78
2	24.55	Benzoic acid, 2-(methylamino)-, methyl ester	1.66
3	13.84	d-Limonene	1.44
4	18.35	4-Terpineol	1.30
5	26.60	d-Germacrene	1.25
6	15.98	2-Cyclohexen-1-ol, 1-methyl-4-(1-methylethyl)-, cis-	1.25
7	24.31	β-Cubebene	1.25

## Supplementary Materials

The following materials are available online at http://www.mdpi.com/1420-3049/23/5/1235/s1: Table S1: Identification of volatile compounds by HS-SPME-GC-MS, and Table S2: Details of mature tangerine fruits from various species collected in this work.

## References

[1] State Pharmacopoeia Committee of People’s Republic of China (2015). Pharmacopoeia of People's Republic of China.

[2] Liu E.H., Zhao P., Duan L., Zheng G.D., Guo L., Yang H., Li P. (2013). Simultaneous determination of six bioactive flavonoids in Citri Reticulatae Pericarpium by rapid resolution liquid chromatography coupled with triple quadrupole electrospray tandem mass spectrometry. Food Chem..

[3] Yu X., Sun S., Guo Y., Liu Y., Yang D., Li G., Lü S. (2018). Citri Reticulatae Pericarpium (Chenpi): Botany, ethnopharmacology, phytochemistry, and pharmacology of a frequently used traditional Chinese medicine. J. Ethnopharmacol..

[4] Zhang L., Liu Y., Liu Z., Wang C., Song Z., Liu Y., Dong Y., Ning Z., Lu A. (2016). Comparison of the roots of *Salvia miltiorrhiza* Bunge (Danshen) and its variety *S. miltiorrhiza* Bge f. Alba (*Baihua Danshen*) based on multi-wavelength HPLC-fingerprinting and contents of nine active components. Anal. Methods.

[5] Fan X.-H., Cheng Y.-Y., Ye Z.-L., Lin R.-C., Qian Z.-Z. (2006). Multiple chromatographic fingerprinting and its application to the quality control of herbal medicines. Anal. Chim. Acta.

[6] Lu G.-H., Chan K., Liang Y.-Z., Leung K., Chan C.-L., Jiang Z.-H., Zhao Z.-Z. (2005). Development of high-performance liquid chromatographic fingerprints for distinguishing Chinese Angelica from related umbelliferae herbs. J. Chromatogr. A.

[7] Wang D., Wang J., Huang X., Tu Y., Ni K. (2007). Identification of polymethoxylated flavones from green tangerine peel (Pericarpium Citri Reticulatae Viride) by chromatographic and spectroscopic techniques. J. Pharm. Biomed. Anal..

[8] Yang Y., Zhao X.J., Pan Y., Zhou Z. (2016). Identification of the chemical compositions of Ponkan peel by ultra performance liquid chromatography coupled with quadrupole time-of-flight mass spectrometry. Anal. Methods.

[9] Xing T.T., Zhao X.J., Zhang Y.D., Li Y.F. (2017). Fast Separation and Sensitive Quantitation of Polymethoxylated Flavonoids in the Peels of Citrus Using UPLC-Q-TOF-MS. J. Agric. Food Chem..

[10] Sawamura M., Thi Minh Tu N., Onishi Y., Ogawa E., Choi H.S. (2004). Characteristic odor components of Citrus reticulata Blanco (ponkan) cold-pressed oil. Biosci. Biotechnol. Biochem..

[11] Yi L., Dong N., Liu S., Yi Z., Zhang Y. (2015). Chemical features of Pericarpium Citri Reticulatae and Pericarpium Citri Reticulatae Viride revealed by GC-MS metabolomics analysis. Food Chem..

[12] Chen M.H., Huang T.C. (2016). Volatile and Nonvolatile Constituents and Antioxidant Capacity of Oleoresins in Three Taiwan Citrus Varieties as Determined by Supercritical Fluid Extraction. Molecules.

[13] Merkle S., Kleeberg K., Fritsche J. (2015). Recent Developments and Applications of Solid Phase Microextraction (SPME) in Food and Environmental Analysis—A Review. Chromatography.

[14] Balasubramanian S., Panigrahi S. (2010). Solid-Phase Microextraction (SPME) Techniques for Quality Characterization of Food Products: A Review. Food Bioprocess Technol..

[15] State Pharmacopoeia Committee of People’s Republic of China (2015). Pharmacopoeia of the People's Republic of China.

[16] Ai J. (1997). Headspace Solid Phase Microextraction. Dynamics and Quantitative Analysis before Reaching a Partition Equilibrium. Anal. Chem..

[17] Ai J. (1997). Solid Phase Microextraction for Quantitative Analysis in Nonequilibrium Situations. Anal. Chem..

[18] Zhang J., Li L., Gao N., Wang D., Gao Q., Jiang S. (2010). Feature extraction and selection from volatile compounds for analytical classification of Chinese red wines from different varieties. Anal. Chim. Acta.

[19] Andersen N.H., Syrdal D.D. (1970). Terpenes and sesquiterpenes of chamaecyparis nootkatensis leaf oil Phytochemistry. Phytochemistry.

[20] Jr P.C.H., Pitzer E.W. (1982). Characterizing petroleum- and shale-derived jet fuel distillates via temperature-programmed Kováts indices. J. Chromatogr. A.

[21] He M., Yan P., Yang Z.Y., Zhang Z.M., Yang T.B., Hong L. (2018). A modified multiscale peak alignment method combined with trilinear decomposition to study the volatile/heat-labile components in Ligusticum chuanxiong Hort—Cyperus rotundus rhizomes by HS-SPME-GC/MS. J. Chromatogr. B.

[22] Muhammad M., Mueen I., Muhammad A., Muhammad S., Summar A., Muhammad S., Muhammad J. (2012). Antioxidant and Antipathogenic Activities of Citrus Peel Oils. J. Essent. Oil Bear. Plants.

[23] Yin C., Liu W., Li Z., Pan Z., Lin T., Zhang M. (2015). Chemometrics to chemical modeling: Structural coding in hydrocarbons and retention indices of gas chromatography. J. Sep. Sci..

[24] Kim T.H., Thuy N.T., Shin J.H., Baek H.H., Lee H.J. (2000). Aroma-active compounds of miniature beefsteakplant (Mosla dianthera Maxim). J. Agric. Food Chem..

[25] Malingré T.M., Maarse H. (1974). Composition of the essential oil of Mentha aquatica. Phytochemistry.

[26] Palá-Paúl J., Pérez-Alonso M.J., Velasco-Negueruela A., Ramos-Vázquez P., Gómez-Contreras F., Sanz J. (2015). Essential oil of *Santolina rosmarinifolia* L. ssp. rosmarinifolia: First isolation of capillene, a diacetylene derivative. Flavour Fragr. J..

[27] Kim T.H., Lee S.M., Kim Y.S., Kim K.H., Oh S., Lee H.J. (2003). Aroma dilution method using GC injector split ratio for volatile compounds extracted by headspace solid phase microextraction. Food Chem..

[28] Choi H.S. (2003). Character impact odorants of citrus hallabong ([C. unshiu Marcov x C. sinensis Osbeck] x C. reticulata Blanco) cold-pressed peel oil. J. Agric. Food Chem..

[29] Shellie R., Mondello L., Marriott P., Dugo G. (2002). Characterisation of lavender essential oils by using gas chromatography-mass spectrometry with correlation of linear retention indices and comparison with comprehensive two-dimensional gas chromatography. J. Chromatogr. A.

[30] Khan M., Mahmood A., Alkhathlan H.Z. (2016). Characterization of leaves and flowers volatile constituents of Lantana camara growing in central region of Saudi Arabia. Arab. J. Chem..

[31] Thomas A.F., Bassols F. (1992). Occurrence of pyridines and other bases in orange oil. J. Agric. Food Chem..

[32] Sugisawa H., Yang R.H., Kawabata C., Tamura H. (1989). Volatile Constituents in the Peel Oil of Sudachi (*Citrus sudachi*). Agric. Biol. Chem..

[33] Shen Y., Hou J., Deng W., Feng Z., Yang M., Cheng J., Wu W., Guo D.A. (2016). Comparative Analysis of Ultrafine Granular Powder and Decoction Pieces of Salvia miltiorrhiza by UPLC-UV-MSn Combined with Statistical Analysis. Planta Med..

[34] Bylesjö M., Rantalainen M., Cloarec O., Nicholson J.K., Holmes E., Trygg J. (2006). OPLS discriminant analysis: Combining the strengths of PLS-DA and SIMCA classification. J. Chemom..

[35] Huang B.M., Zha Q.L., Chen T.B., Xiao S.Y., Xie Y., Luo P., Wang Y.P., Liu L., Zhou H. (2018). Discovery of markers for discriminating the age of cultivated ginseng by using UHPLC-QTOF/MS coupled with OPLS-DA. Phytomedicine.

