# Supplementary material for: Study on the Discrimination between Citri Reticulatae Pericarpium Varieties Based on HS-SPME-GC-MS Combined with Multivariate Statistical Analyses
Source: Molecules. 2018 May 22;23(5):1235. doi: 10.3390/molecules23051235 (PMC6099961; doi:10.3390/molecules23051235)
Supplement: Supplementary file 1 [file molecules-23-01235-s001.pdf]

Table S1. Identification of volatile compounds by HS-SPME-GC-MS.

No.	Compounds	CAS number	Molecular formula	RT [a] (min)	LRI[b]	LRI[c]	Relative areas [d]										
							GCP	CP1	CP2	CP3	CP4	CP5	CP6	CP7	CP8	CP9	CP10
1	α-Thujene	2867-05-2	$C_{10}H_{16}$	10.60	929	924	0.067	0.060	0.075	0.110	0.074	nd	0.031	0.041	0.031	0.037	0.297
2	α-Pinene	7785-70-8	$C_{10}H_{16}$	10.83	936	948	0.264	0.432	0.288	0.662	0.414	0.215	0.272	0.338	0.625	0.404	0.050
3	β-Thujene	28634-89-1	$C_{10}H_{16}$	12.08	973	968	0.047	0.054	0.065	0.219	0.070	0.046	0.215	0.073	0.329	0.047	0.058
4	β-Pinene	18172-67-3	$C_{10}H_{16}$	12.19	977	987	0.252	0.270	0.215	0.397	0.254	0.134	0.087	0.443	0.090	0.312	0.200
5	β-Myrcene	123-35-3	$C_{10}H_{16}$	12.62	990	993	0.346	0.948	0.667	1.634	1.052	0.549	0.831	0.037	3.552	0.527	1.408
6	3-Carene	13466-78-9	$C_{10}H_{16}$	13.46	1016	1013	0.059	0.059	0.041	0.128	0.054	0.010	0.038	0.044	0.034	0.036	0.150
7	o-Cymene	527-84-4	$C_{10}H_{14}$	13.65	1022	1025	1.327	0.758	1.085	1.280	1.193	0.560	0.656	1.472	0.475	0.944	1.055
8	D-Limonene	5989-27-5	$C_{10}H_{16}$	13.84	1028	1028	24.286	48.863	43.325	76.515	45.072	38.470	44.514	45.931	130.802	39.245	56.755
9	γ-Terpinene	99-85-4	$C_{10}H_{16}$	14.77	1058	1053	8.553	6.590	5.671	11.909	6.029	3.118	2.475	6.070	4.582	5.773	7.527
10	trans-4-Thujanol	17699-16-0	$C_{10}H_{18}O$	15.04	1066	1081	0.974	0.337	0.803	0.449	0.500	0.441	0.445	0.423	0.234	0.418	0.254
11	Terpinolene	586-62-9	$C_{10}H_{16}$	15.71	1088	1083	0.458	0.358	0.325	0.752	0.306	0.175	0.148	0.409	0.312	0.347	0.531
12	2-Cyclohexen-1-ol, 1-methyl-4-(1-methylethyl)-, cis-	29803-82-5	$C_{10}H_{18}O$	15.98	1096	1092	5.507	9.430	11.426	10.092	11.187	16.298	15.768	17.103	3.902	17.502	7.707
3	trans-p-Mentha-2,8-dienol	3886-78-0	$C_{10}H_{16}O$	16.64	1119	1120	0.900	0.351	0.973	0.669	0.832	0.419	0.734	0.903	0.768	0.281	0.429
14	2-Cyclohexen-1-ol, 1-methyl-4-(1-methylethenyl)-, trans-	7212-40-0	$C_{10}H_{16}O$	17.08	1134	1142	0.502	nd	0.222	0.201	0.221	0.191	0.104	0.270	nd	nd	0.215
15	Bicyclo[3.1.0]hexan-2-ol, 2-methyl-5-(1-methylethyl)-, (1α,2α,5α)-	17699-16-0	$C_{10}H_{18}O$	17.26	1140	1141	0.071	nd	0.024	0.022	0.013	nd	nd	0.017	nd	nd	nd
16	L-camphor	464-49-3	$C_{10}H_{16}O$	17.34	1142	1144	0.059	nd	0.028	0.018	0.010	nd	nd	nd	nd	nd	nd
17	6-Octenal, 3,7-dimethyl-, (R)-	106-23-0	$C_{10}H_{18}O$	17.50	1148	1152	0.155	0.015	0.118	0.169	0.039	nd	0.088	0.424	nd	0.259	nd
18	Bicyclo[2.2.1]heptan-2-ol, 1,7,7-trimethyl-, (1S-endo)-	464-45-9	$C_{10}H_{18}O$	18.08	1167	1160	0.085	nd	0.013	nd	nd	nd	nd	nd	nd	nd	nd

No.	Compounds	CAS number	Molecular formula	RT [a] (min)	LRI[b]	LRI[c]	Relative areas [d]										
							GCP	CP1	CP2	CP3	CP4	CP5	CP6	CP7	CP8	CP9	CP10
19	4-Terpineol	562-74-3	$C_{10}H_{18}O$	18.35	1177	1179	2.687	0.695	2.124	1.789	1.485	1.018	1.191	1.316	1.070	0.853	0.687
20	p-Cymen-8-ol	1197-01-9	$C_{10}H_{14}O$	18.49	1181	1181	0.894	0.115	0.357	0.353	0.319	0.114	0.201	0.268	0.336	0.090	0.239
21	Cyclohexanol, 2-methylene-5-(1-methylethenyl)-	35907-10-9	$C_{10}H_{16}O$	18.64	1186	1186	0.403	0.070	0.147	0.159	0.217	0.067	0.115	0.064	0.141	0.030	0.045
22	α-Terpineol	98-55-5	$C_{10}H_{18}O$	18.72	1189	1185	10.217	4.107	10.065	7.359	7.328	6.237	7.127	6.828	5.854	5.530	3.921
23	Decanal	112-31-2	$C_{10}H_{20}O$	19.02	1199	1197	1.211	0.980	1.209	1.383	1.661	1.356	1.972	3.430	1.448	2.283	0.641
24	cis-Carveol	1197-06-4	$C_{10}H_{16}O$	19.52	1217	1214	0.873	0.329	0.626	0.614	0.741	0.494	0.765	0.459	0.735	0.484	0.359
25	L-Carveol	99-48-9	$C_{10}H_{16}O$	19.6	1220	1220	0.379	0.018	0.110	0.080	0.024	nd	0.016	0.245	nd	nd	nd
26	Isogeraniol	5944-20-7	$C_{10}H_{18}O$	19.75	1226	1228	1.397	0.291	0.654	0.772	0.493	0.504	1.016	0.704	1.245	nd	0.426
27	β-Citronellol	106-22-9	$C_{10}H_{20}O$	19.85	1229	1229	0.524	0.164	0.030	1.098	0.340	0.239	0.261	0.782	0.333	nd	0.195
28	D-Carvone	2244-16-8	$C_{10}H_{14}O$	20.14	1240	1240	0.435	0.238	0.460	0.378	0.364	0.349	0.491	0.435	0.345	0.438	0.164
29	Piperitone	89-81-6	$C_{10}H_{16}O$	20.48	1252	1253	0.180	0.114	0.137	0.096	0.071	0.181	0.067	0.138	0.063	0.203	nd
30	Perilla aldehyde	2111-75-3	$C_{10}H_{14}O$	21.00	1271	1271	1.338	1.017	1.873	1.522	1.544	1.728	1.526	1.583	1.319	1.805	0.664
31	Thymol	89-83-8	$C_{10}H_{14}O$	21.40	1285	1283	0.583	0.098	0.092	0.115	0.073	0.016	0.016	0.170	0.060	nd	0.165
32	Carvacrol	499-75-2	$C_{10}H_{14}O$	21.57	1291	1297	3.345	0.650	3.806	1.636	0.753	0.537	0.690	9.240	0.936	0.104	1.556
33	Methyl caprate	110-42-9	$C_{11}H_{22}O_2$	22.31	1319	1316	0.184	0.122	0.429	0.167	0.133	0.128	0.220	0.468	0.491	0.131	0.204
34	Methyl anthranilate	134-20-3	$C_8H_9NO_2$	22.79	1338	1332	0.155	0.005	0.027	0.071	nd	0.014	nd	nd	0.075	nd	nd
35	Citronellol acetate	150-84-5	$C_{12}H_{22}O_2$	23.06	1348	1348	0.233	0.275	0.294	0.334	0.243	0.693	0.525	0.494	0.359	0.591	0.197
36	α-Cubebene	17699-14-8	$C_{15}H_{24}$	23.24	1355	1354	0.176	0.394	0.307	0.204	0.308	0.322	0.694	0.478	0.102	0.802	0.292
37	Neryl acetate	141-12-8	$C_{12}H_{20}O_2$	23.33	1358	1356	0.356	0.710	0.697	0.727	0.781	1.947	2.612	1.565	0.262	2.255	0.491
38	Copaene	3856-25-5	$C_{15}H_{24}$	23.97	1383	1378	1.016	1.600	1.511	0.904	1.272	1.532	2.623	2.387	0.459	3.797	0.977
39	β-Cubebene	13744-15-5	$C_{15}H_{24}$	24.31	1396	1394	0.699	4.951	2.311	1.229	1.556	2.432	3.701	3.344	0.725	9.178	2.073
40	Benzoic acid, 2-(methylamino)-, methyl ester	85-91-6	$C_9H_{11}NO_2$	24.55	1406	1402	40.066	5.184	28.840	19.674	17.169	11.435	12.395	9.195	27.093	6.574	7.031
41	Caryophyllene	87-44-5	$C_{15}H_{24}$	25.11	1428	1427	3.411	1.377	3.087	1.321	0.869	1.102	1.983	2.049	0.749	2.867	0.507
42	(Z)-β-Farnesene	28973-97-9	$C_{15}H_{24}$	25.80	1457	1458	0.095	0.216	0.823	0.425	0.144	0.316	1.051	0.677	0.268	0.696	0.173
43	Germacrene D	23986-74-5	$C_{15}H_{24}$	26.60	1489	1488	0.505	4.142	2.308	1.410	1.165	2.200	3.425	5.788	0.772	7.269	1.736
44	(3Z,6E)- α-Farnesene	26560-14-5	$C_{15}H_{24}$	26.76	1496	1496	0.134	0.919	0.189	0.138	0.061	0.262	0.314	0.304	nd	1.812	0.570
45	α-Farnesene	502-61-4	$C_{15}H_{24}$	27.03	1507	1503	11.411	8.944	9.929	5.120	4.378	6.185	8.240	23.732	4.873	19.305	4.650

No.	Compounds	CAS number	Molecular formula	RT [a] (min)	LRI[b]	LRI[c]	Relative areas [d]										
							GCP	CP1	CP2	CP3	CP4	CP5	CP6	CP7	CP8	CP9	CP10
46	δ-Guaiene	3691-11-0	$C_{15}H_{24}$	27.21	1515	1512	0.114	1.848	0.666	0.359	0.274	0.718	1.223	1.439	0.273	4.439	0.628
47	Dodecanoic acid, methyl ester	111-82-0	$C_{13}H_{26}O_2$	27.34	1520	1520	0.140	0.327	0.469	0.297	0.101	0.199	0.482	0.537	0.575	0.437	0.177
48	Cadinene	523-47-7	$C_{15}H_{24}$	27.55	1529	1529	1.713	3.368	2.718	1.515	2.099	2.564	5.401	3.734	1.032	7.382	1.634
49	Caryophyllene oxide	1139-30-6	$C_{15}H_{24}O$	28.99	1591	1596	0.142	0.046	0.137	0.023	0.019	0.039	0.187	0.174	0.071	0.088	0.100
50	α-Sinensal	17909-77-2	$C_{15}H_{22}O$	32.48	1752	1752	0.828	nd	0.160	0.072	0.028	0.053	0.209	nd	0.240	nd	nd
51	Hexadecanoic acid, methyl ester	112-39-0	$C_{17}H_{34}O_2$	35.85	1924	1924	0.085	nd	0.035	0.028	0.017	nd	0.103	0.059	0.179	nd	nd

[a] Retention time (min). [b] Linear retention indices of the analyte. [c] Linear retention indices reported in the database. [d] Peak area ratios relative to n-tridecan (internal standard). nd, not detected. Nd, not detected.

GCP, *Citrus reticulata* 'Chachi' (n=31).CP1, *C. reticulata* 'Unshiu'(n=3). CP2, *C. reticulata* 'Zhuhong' (n=3). CP3, *C. reticulata* 'Ponkan' (n=5). CP4, *C. reticulata* 'Suavissima' (n=3). CP5, *C. reticulata* 'Tankan' (n=2). CP6, *C. reticulata* 'Hanggan' (n=2). CP7, *C. reticulata* 'Kinokuni' (n=2). CP8, *C. reticulata* 'Shiyue Ju' (n=1). CP9, *C. reticulata* 'Subcompressa' (n=1). CP10, *C. cavaleriei* H. Léveillé ex Cavalerie (n=1).

Table S2. Details of mature tangerine fruits of different species collected in this work.

Sample No.	Scientific species name	Classification	Place of collection
S1	*Citrus reticulata* 'Unshiu'	CP	Taizhou, Fujian
S2	*C. reticulata* 'Unshiu'	CP	Taizhou, Fujian
S3	*C. reticulata* 'Unshiu'	CP	Yichang, Hubei
S4	*C. reticulata* 'Zhuhong'	CP	Ji'an, Jiangxi
S5	*C. reticulata* 'Zhuhong'	CP	Ji'an, Jiangxi
S6	*C. reticulata* 'Zhuhong'	CP	Chongqing
S7	*C. reticulata* 'Ponkan'	CP	Ji'an, Jiangxi
S8	*C. reticulata* 'Ponkan'	CP	Chongqing
S9	*C. reticulata* 'Ponkan'	CP	Chongqing
S10	*C. reticulata* 'Ponkan'	CP	Quanzhou, Fujian
S11	*C. reticulata* 'Ponkan'	CP	Quzhou, Zhejiang
S12	*C. reticulata* 'Suavissima'	CP	Wenzhou, Zhejiang
S13	*C. reticulata* 'Suavissima'	CP	Wenzhou, Zhejiang
S14	*C. reticulata* 'Suavissima'	CP	Wenzhou, Zhejiang
S15	*C. reticulata* 'Tankan'	CP	Chaozhou, Guangdong
S16	*C. reticulata* 'Tankan'	CP	Chaozhou, Guangdong
S17	*C. reticulata* 'Hanggan'	CP	Shaoguan, Guangdong
S18	*C. reticulata* 'Hanggan'	CP	Shaoguan, Guangdong
S19	*C. reticulata* 'Kinokuni'	CP	Guangxi
S20	*C. reticulata* 'Kinokuni'	CP	Fuzhou, Jiangxi
S21	*C. reticulata* 'Shiyue Ju'	CP	Sihui, Guangdong
S22	*C. reticulata* 'Subcompressa'	CP	Yongshun County, Hu'nan
S23	*C. cavaleriei H. Léveillé ex Cavalerie*	CP	Yichang, Hubei
G1	*C. reticulata* 'Chachi'	GCP	Xinhui County, Guangdong
G2	*C. reticulata* 'Chachi'	GCP	Xinhui County, Guangdong
G3	*C. reticulata* 'Chachi'	GCP	Xinhui County, Guangdong
G4	*C. reticulata* 'Chachi'	GCP	Xinhui County, Guangdong
G5	*C. reticulata* 'Chachi'	GCP	Xinhui County, Guangdong
G6	*C. reticulata* 'Chachi'	GCP	Xinhui County, Guangdong
G7	*C. reticulata* 'Chachi'	GCP	Xinhui County, Guangdong
G8	*C. reticulata* 'Chachi'	GCP	Xinhui County, Guangdong
G9	*C. reticulata* 'Chachi'	GCP	Xinhui County, Guangdong
G10	*C. reticulata* 'Chachi'	GCP	Xinhui County, Guangdong
G11	*C. reticulata* 'Chachi'	GCP	Xinhui County, Guangdong
G12	*C. reticulata* 'Chachi'	GCP	Xinhui County, Guangdong

G13	*C. reticulata* 'Chachi'	GCP	Xinhui County, Guangdong
G14	*C. reticulata* 'Chachi'	GCP	Xinhui County, Guangdong
G15	*C. reticulata* 'Chachi'	GCP	Xinhui County, Guangdong
G16	*C. reticulata* 'Chachi'	GCP	Xinhui County, Guangdong
G17	*C. reticulata* 'Chachi'	GCP	Xinhui County, Guangdong
G18	*C. reticulata* 'Chachi'	GCP	Xinhui County, Guangdong
G19	*C. reticulata* 'Chachi'	GCP	Xinhui County, Guangdong
G20	*C. reticulata* 'Chachi'	GCP	Xinhui County, Guangdong
G21	*C. reticulata* 'Chachi'	GCP	Xinhui County, Guangdong
G22	*C. reticulata* 'Chachi'	GCP	Xinhui County, Guangdong
G23	*C. reticulata* 'Chachi'	GCP	Xinhui County, Guangdong
G24	*C. reticulata* 'Chachi'	GCP	Xinhui County, Guangdong
G25	*C. reticulata* 'Chachi'	GCP	Xinhui County, Guangdong
G26	*C. reticulata* 'Chachi'	GCP	Xinhui County, Guangdong
G27	*C. reticulata* 'Chachi'	GCP	Xinhui County, Guangdong
G28	*C. reticulata* 'Chachi'	GCP	Xinhui County, Guangdong
G29	*C. reticulata* 'Chachi'	GCP	Xinhui County, Guangdong
G30	*C. reticulata* 'Chachi'	GCP	Xinhui County, Guangdong
G31	*C. reticulata* 'Chachi'	GCP	Xinhui County, Guangdong
